# Supplementary material for: Evidence for a role of human blood-borne factors in mediating age-associated changes in molecular circadian rhythms
Source: bioRxiv. 2024 Jul 1:2023.04.19.537477. Originally published 2023 Apr 20. Preprint. [Version 2] doi: 10.1101/2023.04.19.537477 (PMC10557775; doi:10.1101/2023.04.19.537477)
Supplement: Supplement 1 [file NIHPP2023.04.19.537477v2-supplement-1.pdf]

bioRxiv preprint doi: https://doi.org/10.1101/2023.04.19.537477; this version posted July 1, 2024. The copyright holder for this preprint (which was not certified by peer review) is the author/funder, who has granted bioRxiv a license to display the preprint in perpetuity. It is made available under aCC-BY 4.0 International license.

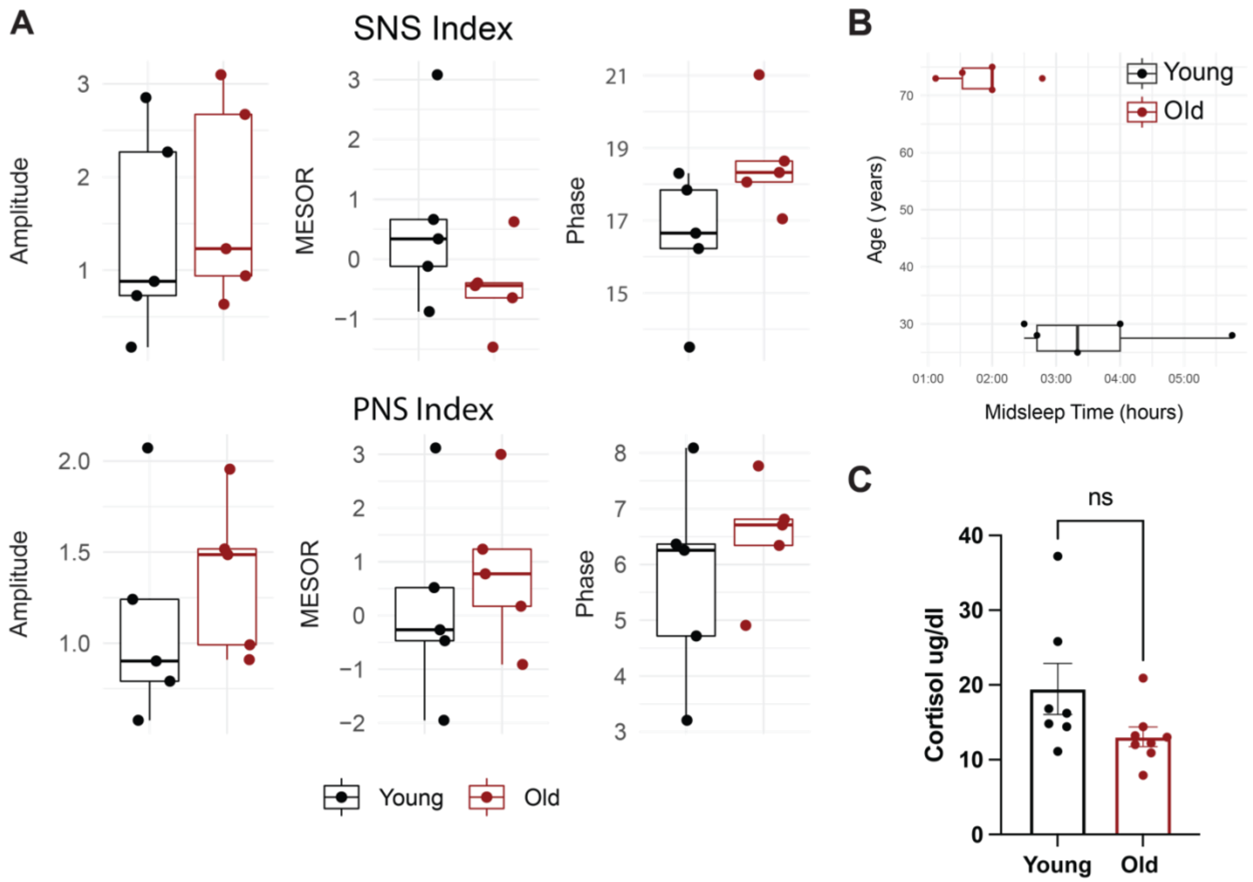

Fig S1. There are differences in midsleep time, but no significant differences in sympathetic, parapsympathetic nervous system indices, or cortisol levels between young and old individuals

Sympathetic nervous system (SNS, A, top) and parasympathetic nervous system (PNS, A, bottom) indices were measured using EKG. Amplitude, MESOR, and Phase were calculated from the resulting oscillations and the Median, quartiles, and SEMs are shown. Dots represent individuals. P>0.05. N=5 per group. MESOR and amplitude were tested using Wilcoxon rank sum exact test, while phase was tested by Kuiper's two-sample test. Boxplot midlines correspond to median values, while the lower and upper hinges correspond to the first and third quartiles, respectively. Boxplot whiskers extend to the smallest/largest points within 1.5 * IQR (inter Quartile Range) of the lower/upper hinge **(A)**. Boxplot distributions of midsleep times for subjects in the young (black) and old (red) cohorts. The midsleep time is advanced in the old

bioRxiv preprint doi: https://doi.org/10.1101/2023.04.19.537477; this version posted July 1, 2024. The copyright holder for this preprint (which was not certified by peer review) is the author/funder, who has granted bioRxiv a license to display the preprint in perpetuity. It is made available under aCC-BY 4.0 International license.

subjects compared to young by Mann-Whitney U test (Wilcoxon rank sum test) p=0.036). We calculated midsleep times for each subject from their responses to the Munich Chronotype Questionnaire (MCTQ). N=5 per group. We were not able to calculate midsleep times for four subjects (two from the old cohort and two from the young cohort) because they used alarms to wake on their non-working days and one old subject because they exhibited a total sleep time less than 6 hours **(B).** Serum cortisol levels are not statistically different by unpaired t-test **(C**, N=7 young, 8 old **)**.

bioRxiv preprint doi: https://doi.org/10.1101/2023.04.19.537477; this version posted July 1, 2024. The copyright holder for this preprint (which was not certified by peer review) is the author/funder, who has granted bioRxiv a license to display the preprint in perpetuity. It is made available under aCC-BY 4.0 International license.

Table S1. Demographic information for subjects in the study.

Age Group	Sex	Age (y)	BMI (kg/m^2)	Total Sleep Time (TST) [h]	Actigraphy	MCTQ	Grip Strength	EKG	RNA-seq: Main	RNA-seq: Pilot (Fig S4)
20-35	Female	25	23.7	6.6	Yes	Yes	Yes	No	No	No
	Female	28	21.2	6.9	Yes	No	Yes	Yes	Yes	No
	Male	28	25	9.7	Yes	Yes	Yes	Yes	Yes	No
	Male	28	25.2	7.4	Yes	No	Yes	No	Yes	No
	Male	28	25.9	9.3	Yes	Yes	Yes	Yes	No	No
	Female	30	28.7	7.4	Yes	Yes	Yes	Yes	Yes	No
	Male	30	25.9	6.4	Yes	Yes	Yes	Yes	No	Yes
Young	***Mean***	***28.1***	***25.1***	***7.7***	—	—	—	—	—	—
	SD	***1.7***	***2.3***	***1.3***	—	—	—	—	—	—
70-85	Male	70	26.8	6.7	Yes	No	Yes	Yes	No	No
	Male	71	26.7	11.3	Yes	Yes	Yes	No	Yes	No
	Female	73	32.4	7.7	Yes	Yes	Yes	No	Yes	No
	Male	73	21.5	6.6	Yes	Yes	Yes	Yes	No	No
	Female	74	26.4	7.5	Yes	No	Yes	No	No	No
	Male	74	27.2	7	Yes	Yes	Yes	Yes	Yes	Yes
	Male	75	28.8	7.8	Yes	Yes	Yes	Yes	No	No
	Female	76	22.3	5.8	Yes	Yes	Yes	Yes	Yes	No
Old	***Mean***	***73.3***	***26.5***	***7.6***	—	—	—	—	—	—
	SD	***2***	***3.4***	***1.7***	—	—	—	—	—	—

Total sleep time (TST) was calculated from ActiLife 6 determined in-bed and out-of-bed times averaged across nights with available actigraphy data (≥7 nights). Old (N=8), Y-young (N=7). For the RNAseq we did a pilot experiment which had one young and one old (Fig S4). The young sample didn't have enough serum left to include that individual in the actual experiment (n=4 per group), but the old subject did. So, one old subject was used in both figures and the young were different.

bioRxiv preprint doi: https://doi.org/10.1101/2023.04.19.537477; this version posted July 1, 2024. The copyright holder for this preprint (which was not certified by peer review) is the author/funder, who has granted bioRxiv a license to display the preprint in perpetuity. It is made available under aCC-BY 4.0 International license.

Figure S2. Subject Inclusion Flow Chart

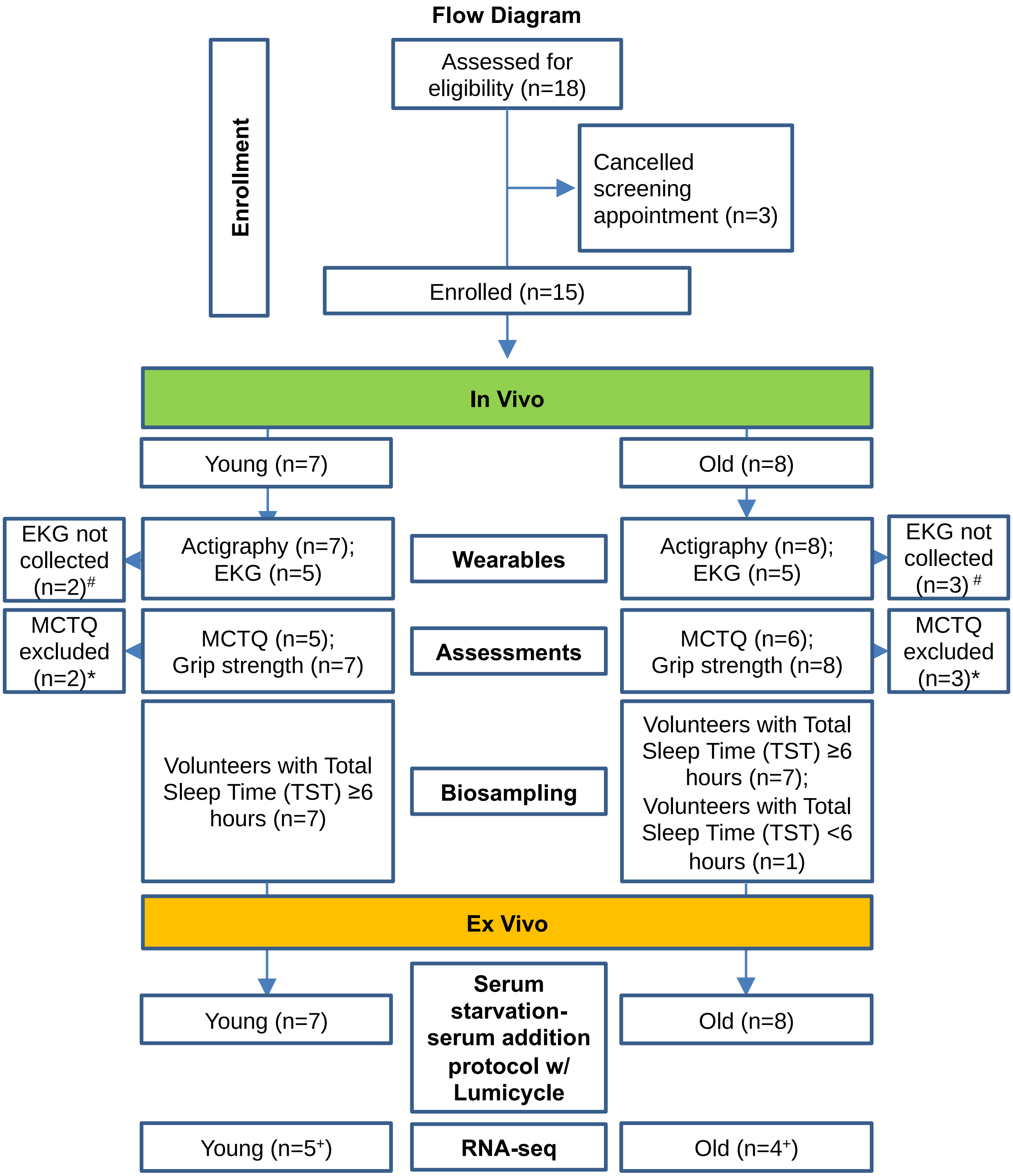

[#] Device did not lock onto an EKG signal
* Use of alarm clocks on non-working days does not allow to calculate chronotype
[+] Due to material limitations, we used sera from two different young subjects for the pilot and main RNA-seq experiments. See Table S1 for further details.

A flow chart of the number of subjects included in each analysis present in this study.

bioRxiv preprint doi: https://doi.org/10.1101/2023.04.19.537477; this version posted July 1, 2024. The copyright holder for this preprint (which was not certified by peer review) is the author/funder, who has granted bioRxiv a license to display the preprint in perpetuity. It is made available under aCC-BY 4.0 International license.

Table S2. BioPatch EKG Data Inclusion Log.

ID	BioPatch EKG data collected?	Reason why BioPatch EKG data was not collected
Old 1	Yes	
Old 2	No	Device was deployed to participant. When device was returned, we found that data had not been collected during the observational period.
Old 3	No	Device was deployed to participant. When device was returned, we found that data had not been collected during the observational period.
Old 4	Yes	
Old 5	Yes	
Old 6	No	Device was deployed to participant. When device was returned, we found that data had not been collected during the observational period.
Old 7	Yes	
Old 8	Yes	
Young 1	Yes	
Young 2	No	Device was deployed to participant. When device was returned, we found that data had not been collected during the observational period.
Young 3	Yes	
Young 4	Yes	
Young 5	Yes	
Young 6	Yes	
Young 7	No	Device was deployed to participant. When device was returned, we found that data had not been collected during the observational period.

A log of which participants were included in our biopatch data based on equipment function.

bioRxiv preprint doi: https://doi.org/10.1101/2023.04.19.537477; this version posted July 1, 2024. The copyright holder for this preprint (which was not certified by peer review) is the author/funder, who has granted bioRxiv a license to display the preprint in perpetuity. It is made available under aCC-BY 4.0 International license.

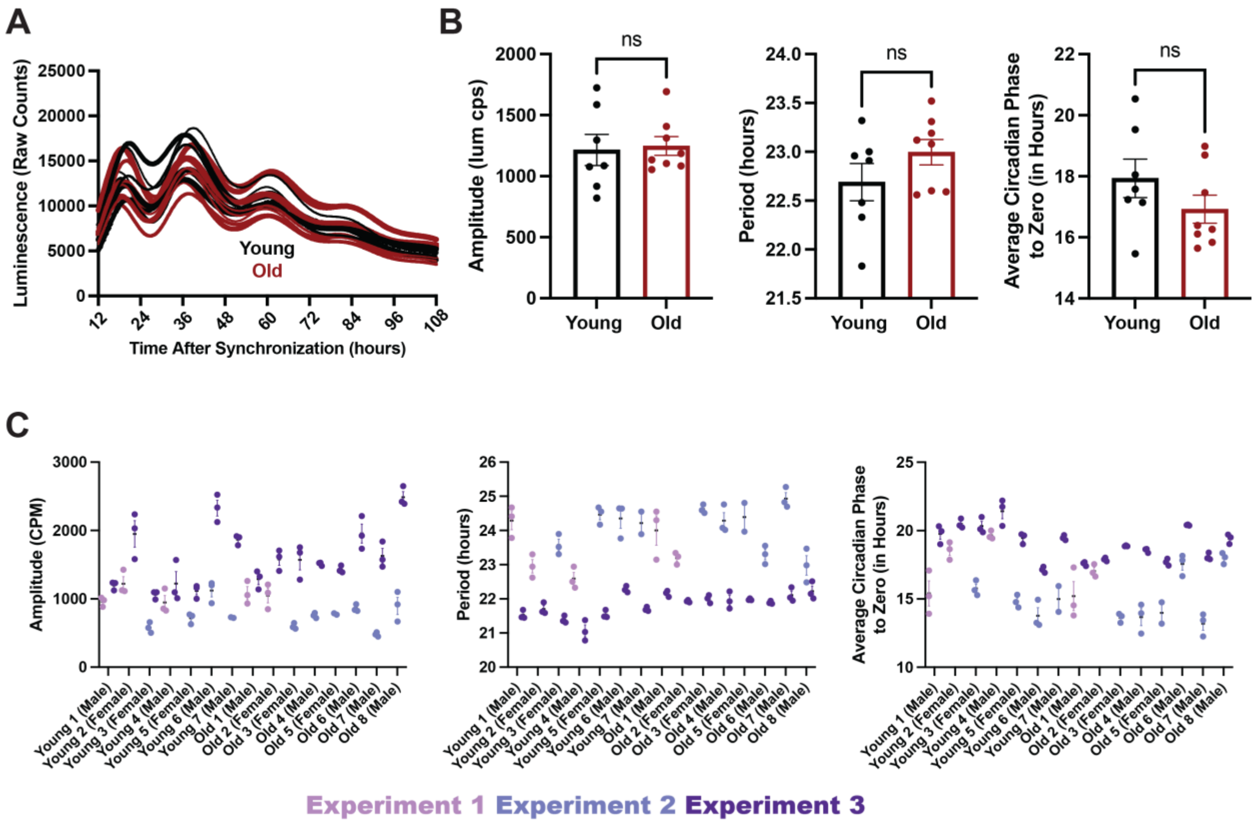

Fig S3: Young and old serum are equally effective at entraining cells in culture

(A,B) Cells synchronized with human serum from either young (N=7 subjects) or old (N=8 subjects) individuals did not show differences in amplitude (as measured in luminescence counts per second, bottom, left), period (bottom, middle), or phase (bottom, right). Line traces and data points represent average values for a specific patient's serum over 2 experiments with 2-3 replicates per experiment. A visual representation of the individual replicates averaged in B **(C)** Summary statistics are displayed as mean +/- SEM. Means compared by unpaired t-test. Despite individual and run to run variability, cells synchronized with human serum from either young (N=7 subjects) or old (N=8 subjects) individuals showed similar amplitude (left), period (middle), and phase relative to synchronization time (right) of BMAL1-luciferase rhythms. Data points represent individual replicates within an experiment. Wells run in the same experiment are displayed in the same color. Each subject's sample was run in 2-3 replicates.

bioRxiv preprint doi: https://doi.org/10.1101/2023.04.19.537477; this version posted July 1, 2024. The copyright holder for this preprint (which was not certified by peer review) is the author/funder, who has granted bioRxiv a license to display the preprint in perpetuity. It is made available under aCC-BY 4.0 International license.

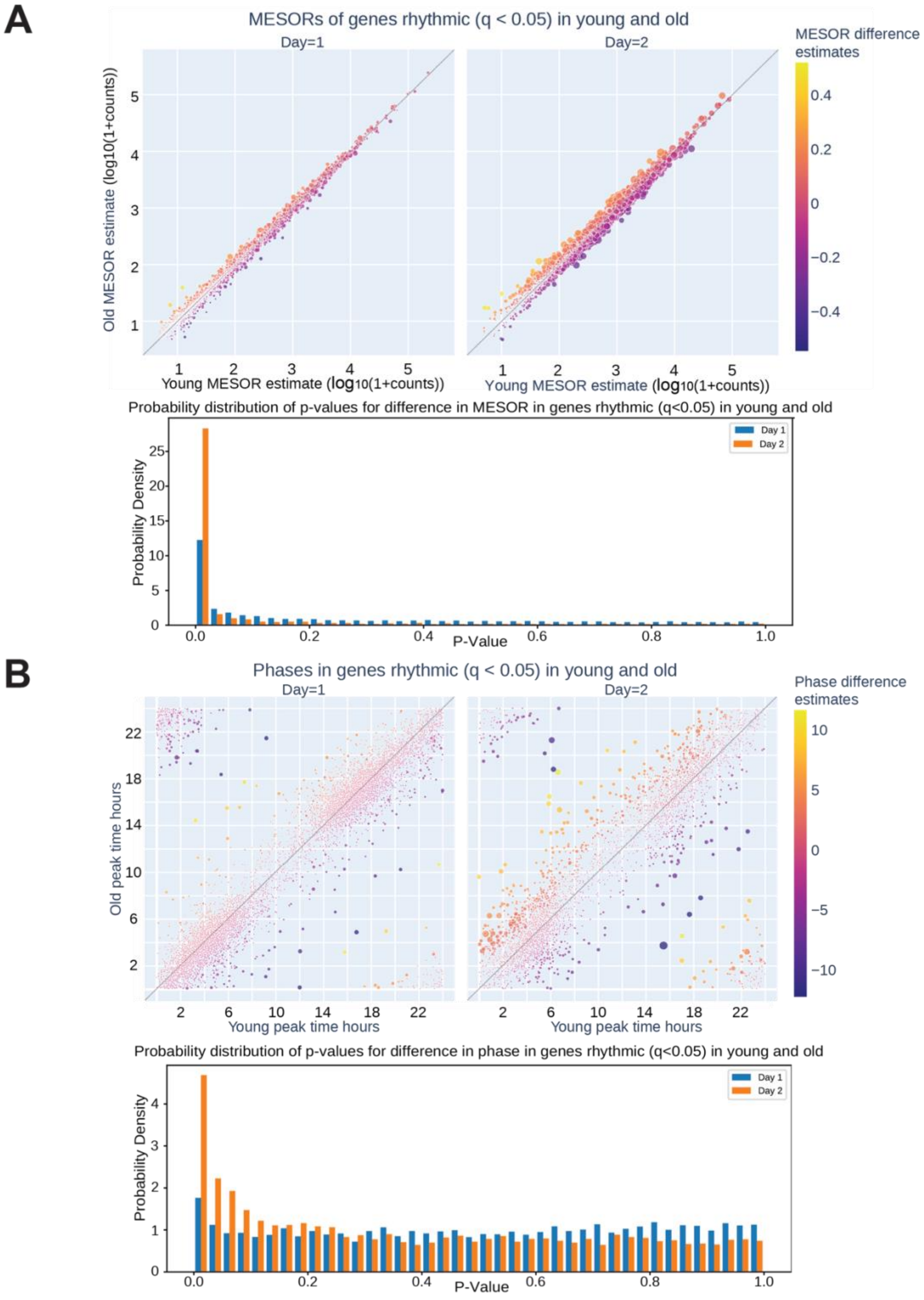

Fig S4. The circadian transcriptomes of cells synchronized with young or old sera deviate significantly on day two.

bioRxiv preprint doi: https://doi.org/10.1101/2023.04.19.537477; this version posted July 1, 2024. The copyright holder for this preprint (which was not certified by peer review) is the author/funder, who has granted bioRxiv a license to display the preprint in perpetuity. It is made available under aCC-BY 4.0 International license.

On day two of serum entrainment, young and old transcriptional rhythms differentiated. CircaCompare analysis of RNA sequencing revealed that on Day 2 of serum entrainment analysis (36-58 hours after synchronization) the MESOR differences of cycling genes are larger between the cells entrained with young or old serum **(A**, top**)**. Additionally, more genes were phase shifted in the old serum condition compared to young serum on Day 2 **(B**, top**)**. The distribution of p-values shows an enrichment of low p-values on Day 2 for both MESOR and phase differences (A, bottom, B, bottom). The size of each circle in the scatter plots is proportional to $-\log_{10}q$, hence bigger circles correspond to smaller q-values for the difference between young and old for each metric.

bioRxiv preprint doi: https://doi.org/10.1101/2023.04.19.537477; this version posted July 1, 2024. The copyright holder for this preprint (which was not certified by peer review) is the author/funder, who has granted bioRxiv a license to display the preprint in perpetuity. It is made available under aCC-BY 4.0 International license.

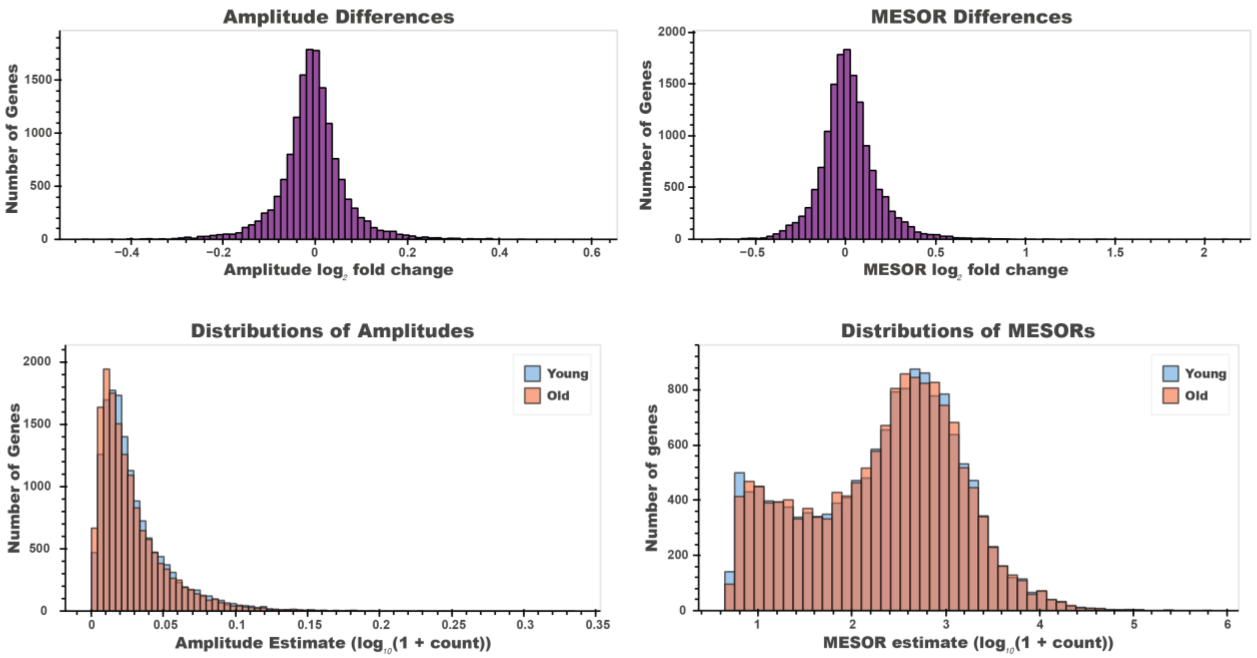

Figure S5 There is a small effect of age on the amplitude and MESOR of cycling mRNA between young and old serum samples. The difference (Top, left) between young and old mRNA amplitude values (bottom, left) are significantly different by Wilcoxon signed-rank test (p= 3.73e-39). The difference (Top, right) between young and old mRNA MESOR values (bottom, right) are significantly different by Wilcoxon signed-rank test (p= 6.94e-22). However, these small differences result in very low p-values due to the large number of genes inspected.

bioRxiv preprint doi: https://doi.org/10.1101/2023.04.19.537477; this version posted July 1, 2024. The copyright holder for this preprint (which was not certified by peer review) is the author/funder, who has granted bioRxiv a license to display the preprint in perpetuity. It is made available under aCC-BY 4.0 International license.

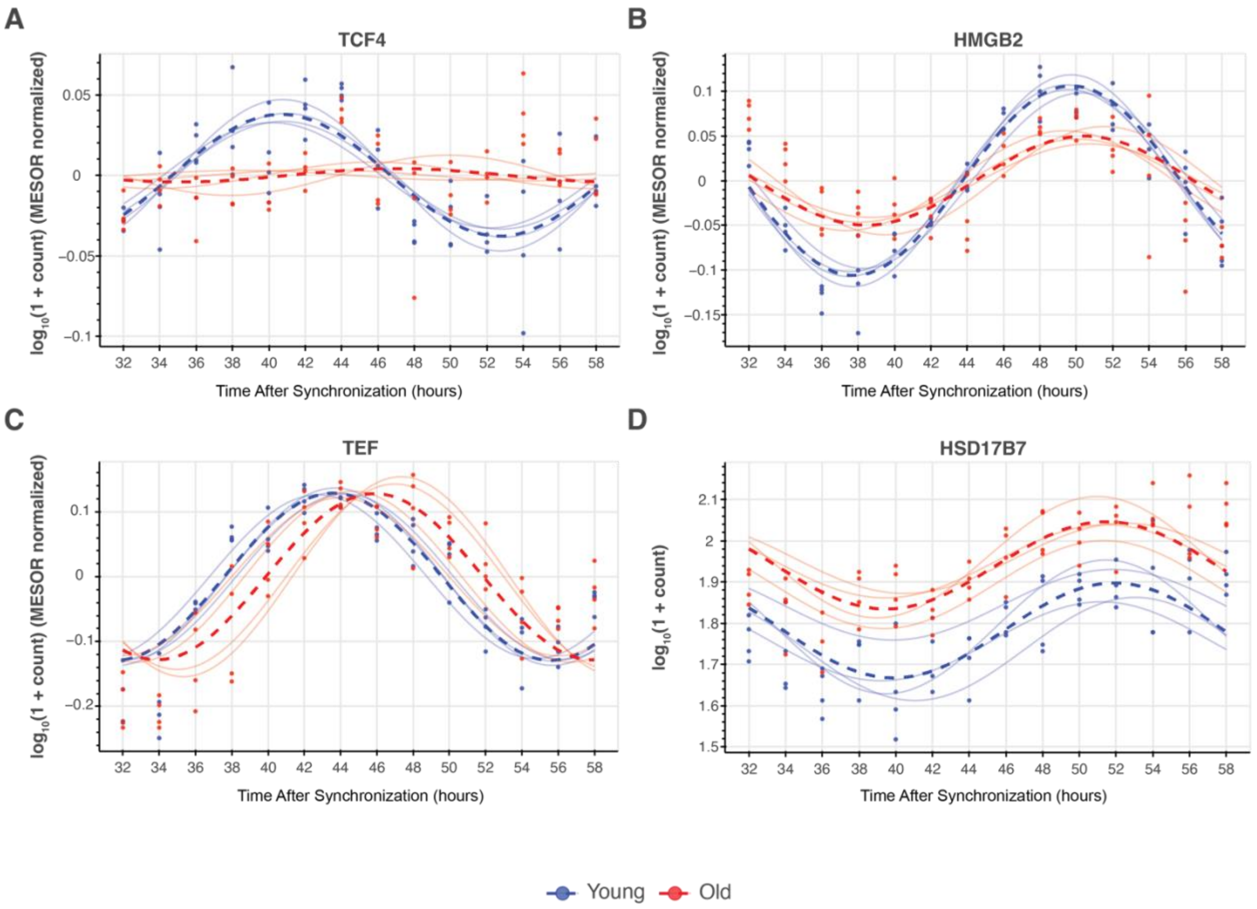

Figure S6: Sample traces of transcripts that cycle with young serum synchronization and are differentially affected by old serum synchronization. Example traces of mRNA transcripts that lose cycling (A, TCF4), decrease amplitude (B, HMGB2), phase shift (C, TEF), and increase MESOR (D, HSD17B7). To ease the comparison of amplitudes and phases in traces depicted in (A-C), subject-level differences in MESORs were removed by subtracting the measured gene expression of each subject at each timepoint by the MESOR of the subject's gene expression oscillation pattern, making all subject-level traces oscillate around zero. Traces in (D) were not subjected to this transformation since (D) showcases differences in MESORs themselves. Transparent lines represent subject-level fits and dashed/opaque lines represent cohort-level fits.

bioRxiv preprint doi: https://doi.org/10.1101/2023.04.19.537477; this version posted July 1, 2024. The copyright holder for this preprint (which was not certified by peer review) is the author/funder, who has granted bioRxiv a license to display the preprint in perpetuity. It is made available under aCC-BY 4.0 International license.

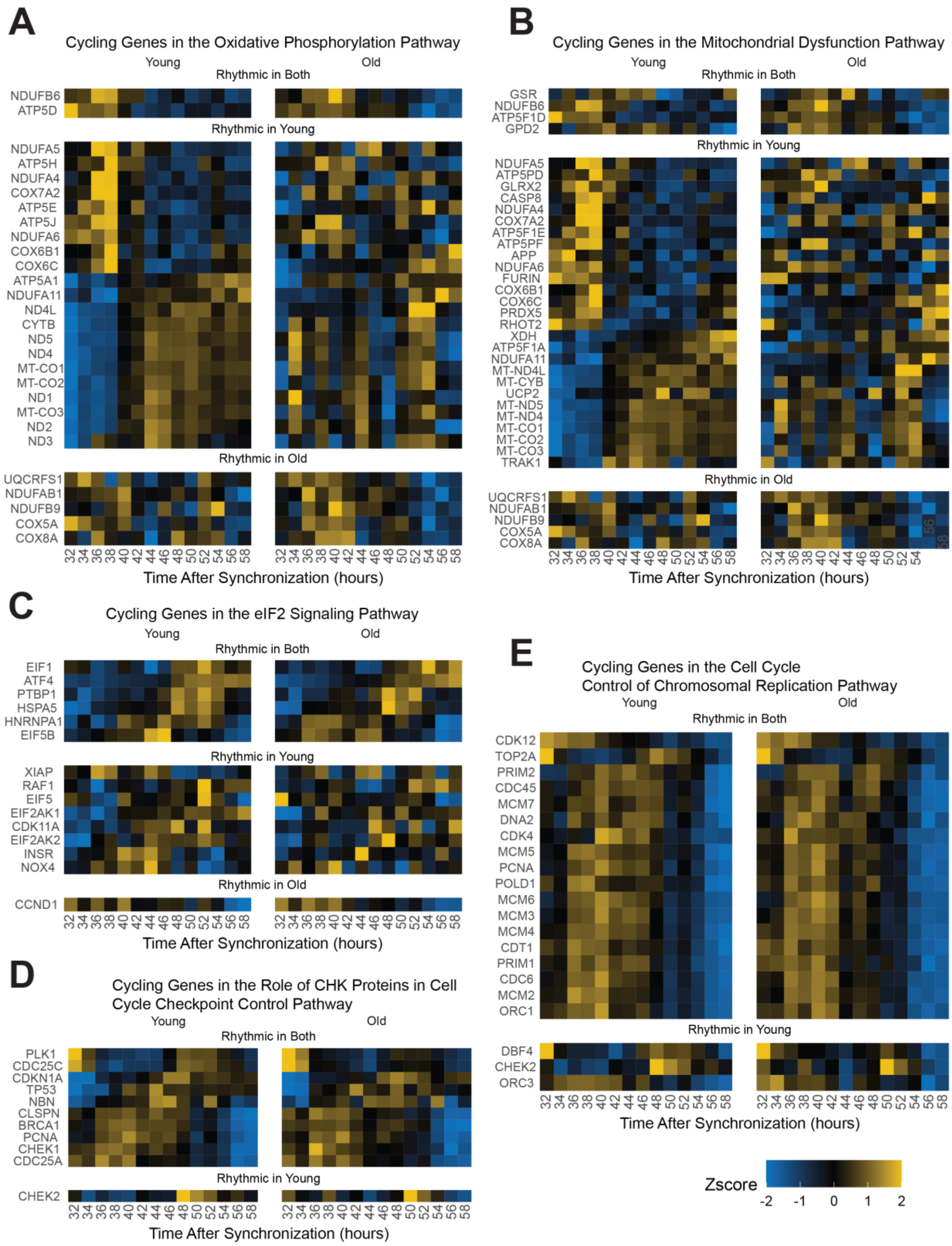

Fig S7: Oxidative phosphorylation genes and chromosomal replication genes are affected by entrainment with old serum

bioRxiv preprint doi: https://doi.org/10.1101/2023.04.19.537477; this version posted July 1, 2024. The copyright holder for this preprint (which was not certified by peer review) is the author/funder, who has granted bioRxiv a license to display the preprint in perpetuity. It is made available under aCC-BY 4.0 International license.

Additional pathway analysis employing IPA further emphasizes finding with STRING analysis. Genes identified by IPA analysis to be associated with oxidative phosphorylation/mitochondrial dysfunction and eIF2 genes lose rhythmicity with age (BIC >0.75) **(A, B, C)**. Cell cycle checkpoint control (CHK proteins) **(D)** and chromosomal replication genes **(E)** maintain their cycling in the aged condition, which is consistent with the continued division of cells.

bioRxiv preprint doi: https://doi.org/10.1101/2023.04.19.537477; this version posted July 1, 2024. The copyright holder for this preprint (which was not certified by peer review) is the author/funder, who has granted bioRxiv a license to display the preprint in perpetuity. It is made available under aCC-BY 4.0 International license.

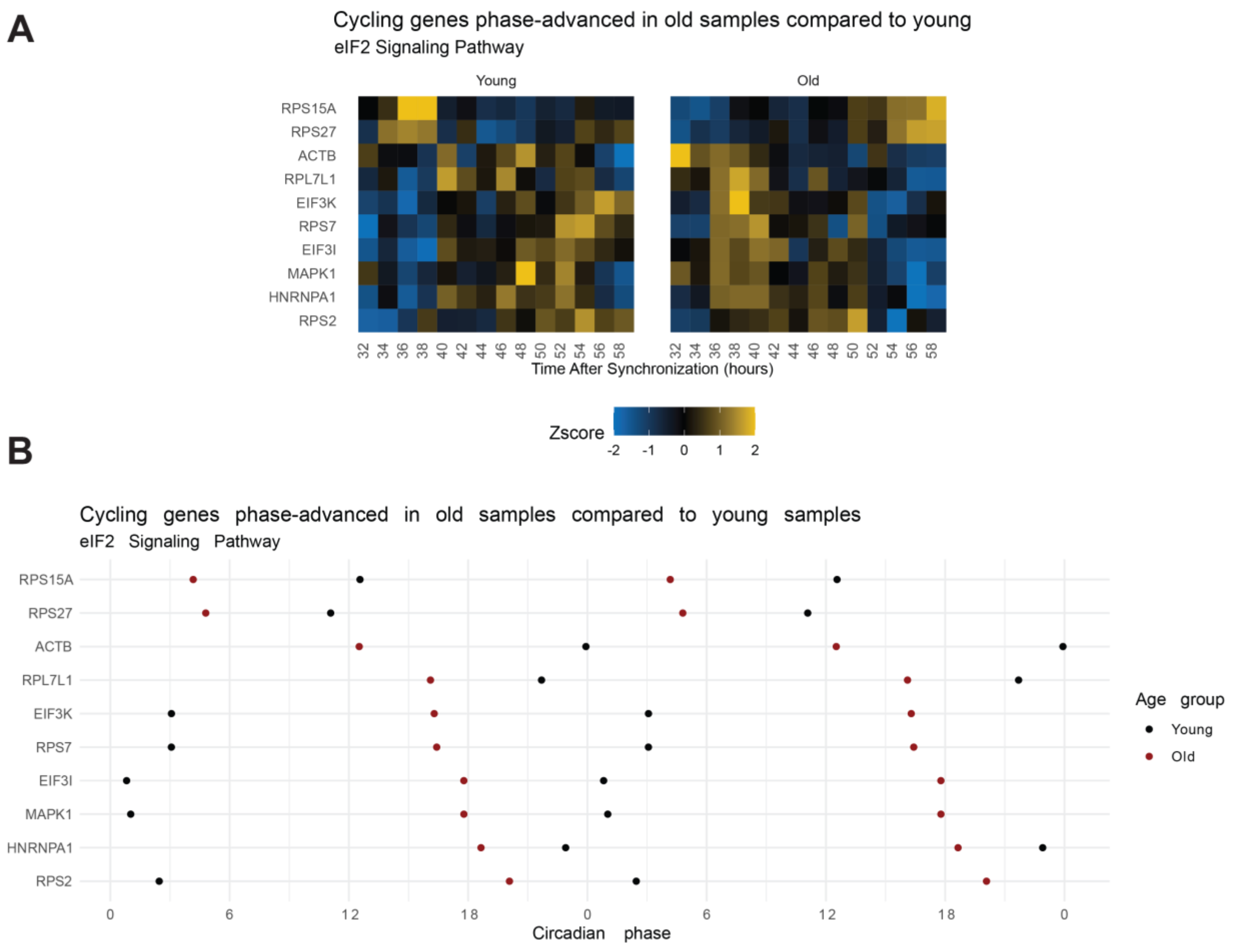

Fig S8: eIF2 signaling pathway genes that maintain cycling with age phase advance in the old serum condition

From the IPA analyses, the eIF2 signaling pathway was enriched (BIC >0.75) in two datasets: 1) genes cycling in the young sera and not the old and 2) cycling genes phase-advanced in the old sera (q<0.05). A significant number of genes that maintain rhythmicity in the old sera condition are phase advanced as visualized in the heat map (A) or plot of peak phase (B). Note, the data in the peak phase plot are double-plotted. This means the range of the x-axis is doubled and the datapoints between 0 and 24 are duplicated in the right half of the plot. Phase is a circular metric meaning a phase of 0 is equivalent to a phase of 24 (1 ⇔ 25, 2 ⇔ 26, etc). Double-plotting is used to visualize data with repeated patterns or when data straddle the 24-0 boundary. As the eIF2 pathway is involved in protein translation, this suggests that the timing of protein translation is phase advanced in the older serum and/or less rhythmic.

bioRxiv preprint doi: https://doi.org/10.1101/2023.04.19.537477; this version posted July 1, 2024. The copyright holder for this preprint (which was not certified by peer review) is the author/funder, who has granted bioRxiv a license to display the preprint in perpetuity. It is made available under aCC-BY 4.0 International license.

Gene	Symbol	Old_mesor_estimate	Young_mesor_estimate
ENSG00000014138	POLA2	1.942220257	2.030676546
ENSG00000062822	POLD1	2.727523056	2.828026817
ENSG00000073111	MCM2	3.003984606	3.115331295
ENSG00000076003	MCM6	3.124116273	3.203138512
ENSG00000085840	ORC1	2.177440998	2.305154417
ENSG00000091651	ORC6	2.415062674	2.513336383
ENSG00000093009	CDC45	2.428834807	2.540049538
ENSG00000094804	CDC6	2.566892644	2.679401971
ENSG00000097046	CDC7	2.279003902	2.377425914
ENSG00000100297	MCM5	3.17577594	3.288560637
ENSG00000101868	POLA1	2.640718445	2.728951631
ENSG00000104738	MCM4	3.310670684	3.413600036
ENSG00000112118	MCM3	3.168296215	3.269321473
ENSG00000117748	RPA2	2.754645539	2.798914718
ENSG00000131747	TOP2A	3.722304752	3.798890616
ENSG00000132383	RPA1	3.238584015	3.287556646
ENSG00000132646	PCNA	3.232760966	3.321439626
ENSG00000135446	CDK4	3.143303063	3.172936739
ENSG00000138346	DNA2	2.113453067	2.238476228
ENSG00000146143	PRIM2	2.620994795	2.675363852
ENSG00000164815	ORC5	2.361538047	2.414675015
ENSG00000166508	MCM7	3.381394362	3.482444492
ENSG00000167258	CDK12	3.10241713	3.129181459
ENSG00000167513	CDT1	2.474002764	2.590319547
ENSG00000198056	PRIM1	2.116803844	2.231962095

mn

Table S3: Genes involved in the IPA cell cycle of chromosome replication pathway show decreased MESOR with age

Table of genes that are rhythmic in both conditions with a decreased MESOR with old serum treatment compared to young serum treatment by CircaCompare. All genes are involved in the cell cycle/DNA replication pathway.

bioRxiv preprint doi: https://doi.org/10.1101/2023.04.19.537477; this version posted July 1, 2024. The copyright holder for this preprint (which was not certified by peer review) is the author/funder, who has granted bioRxiv a license to display the preprint in perpetuity. It is made available under aCC-BY 4.0 International license.

Gene	Symbol	Old_mesor_estimate	Young_mesor_estimate
ENSG00000001630	CYP51A1	2.040377336	1.871207095
ENSG00000079459	FDFT1	3.330291993	3.19001773
ENSG00000104549	SQLE	3.116822033	2.950386727
ENSG00000110921	MVK	2.530172998	2.4121956
ENSG00000112972	HMGCS1	2.93111747	2.696086286
ENSG00000113161	HMGCR	3.011982868	2.833316048
ENSG00000116133	DHCR24	3.506294106	3.343492054
ENSG00000120437	ACAT2	2.71791125	2.58109012
ENSG00000132196	HSD17B7	1.940436315	1.782521965
ENSG00000147383	NSDHL	2.540302415	2.468852126
ENSG00000160285	LSS	3.486802994	3.411431601
ENSG00000160752	FDPS	3.143592562	3.081359846
ENSG00000167508	MVD	2.819963909	2.68386468
ENSG00000172893	DHCR7	2.990620862	2.797567922

Table S4: Genes with increased MESOR in the IPA cholesterol biosynthesis pathway

Table of genes that are rhythmic in both conditions with a decreased MESOR with old serum treatment compared to young serum treatment by CircaCompare. All genes are involved in the cholesterol biosynthesis pathway.
