# Supplementary material for: Evidence for a role of human blood-borne factors in mediating age-associated changes in molecular circadian rhythms
Source: bioRxiv. 2023 Apr 20:2023.04.19.537477. Preprint. [Version 1] doi: 10.1101/2023.04.19.537477 (PMC10557775; doi:10.1101/2023.04.19.537477)
Supplement: Supplementary file 1 [file NIHPP2023.04.19.537477V1-supplement-1.pdf]

bioRxiv preprint doi: https://doi.org/10.1101/2023.04.19.537477; this version posted April 24, 2023. The copyright holder for this preprint (which was not certified by peer review) is the author/funder, who has granted bioRxiv a license to display the preprint in perpetuity. It is made available under aCC-BY 4.0 International license.

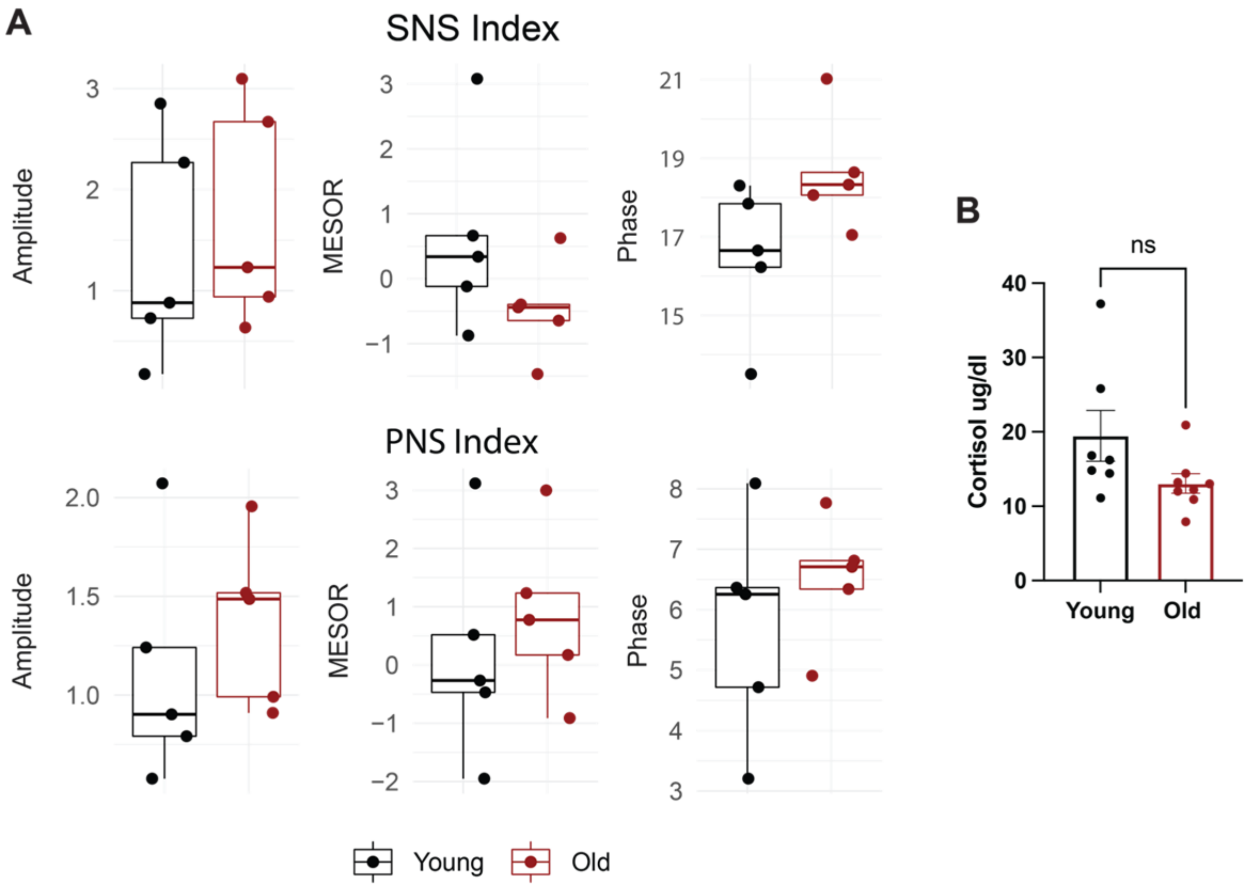

Fig S1. There are no significant differences in grip strength, sympathetic, parapsympathetic nervous system indices, or cortisol levels between young and old individuals

Sympathetic nervous system (SNS, A, top) and parasympathetic nervous system (PNS, A, bottom) indices were measured using EKG. Amplitude, MESOR, and Phase were calculated from the resulting oscillations and the Median, quartiles, and SEMs are shown. Dots represent individuals. P>0.05. N=5 per group. MESOR and amplitude were tested using Wilcoxon rank sum exact test, while phase was tested by Kuiper's two-sample test. Boxplot midlines correspond to median values, while the lower and upper hinges correspond to the first and third quartiles, respectively. Boxplot whiskers extend to the smallest/largest points within 1.5 * IQR (inter Quartile Range) of the lower/upper hinge **(A)**. Serum cortisol levels are not statistically different by unpaired t-test **(B**, N=7 young, 8 old **)**.

bioRxiv preprint doi: https://doi.org/10.1101/2023.04.19.537477; this version posted April 24, 2023. The copyright holder for this preprint (which was not certified by peer review) is the author/funder, who has granted bioRxiv a license to display the preprint in perpetuity. It is made available under aCC-BY 4.0 International license.

Table S1. Demographic information for subjects in the study.

Age Group	Sex	Age (y)	BMI (kg/m^2)	Total Sleep Time (TST) [h]
20-35	Male	30	25.9	6.4
	Female	30	28.7	7.4
	Female	25	23.7	6.6
	Male	28	25.0	9.7
	Female	28	21.2	6.9
	Male	28	25.2	7.4
	Male	28	25.9	9.3
Young	***Mean***	***28.1***	***25.1***	***7.7***
	SD	***1.7***	***2.3***	***1.3***
70-85	Male	76	22.3	5.8
	Male	75	28.8	7.8
	Female	73	32.4	7.7
	Female	74	26.4	7.5
	Male	73	21.5	6.6
	Male	71	26.7	11.3
	Male	74	27.2	7.0
	Male	70	26.8	6.7
Old	***Mean***	***73.3***	***26.5***	***7.6***
	SD	***2.0***	***3.4***	***1.7***

Total sleep time (TST) was calculated from ActiLife 6 determined in-bed and out-of-bed times averaged across nights with available actigraphy data (≥7 nights). Old (N=8), Y-young (N=7).

bioRxiv preprint doi: https://doi.org/10.1101/2023.04.19.537477; this version posted April 24, 2023. The copyright holder for this preprint (which was not certified by peer review) is the author/funder, who has granted bioRxiv a license to display the preprint in perpetuity. It is made available under aCC-BY 4.0 International license.

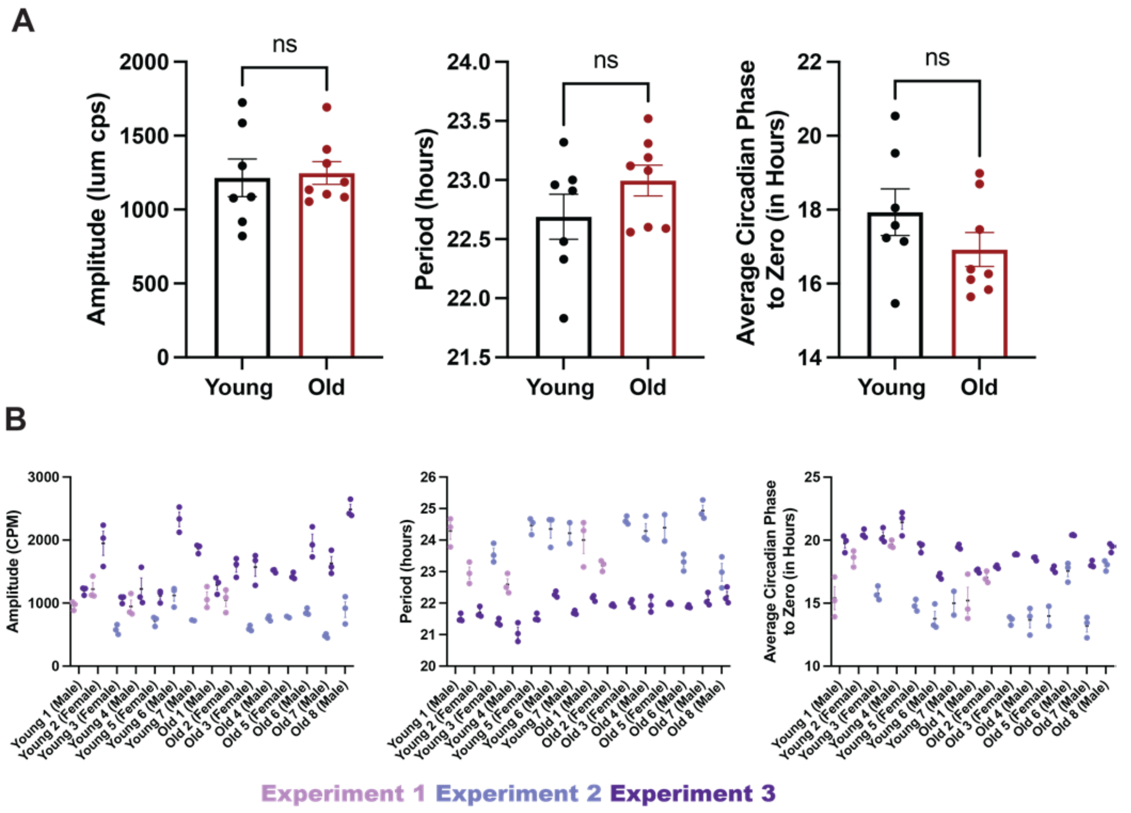

Fig S2: Young and old serum contain equivalent levels of cortisol and are equally effective at entraining cells in culture

(A) Cells synchronized with human serum from either young (N=7 subjects) or old (N=8 subjects) individuals did not show differences in amplitude (as measured in luminescence counts per second, bottom, left), period (bottom, middle), or phase (bottom, right). Data points represent average values for a specific patient's serum over 2 experiments with 2-3 replicates per experiment. A visual representation of the individual replicates averaged in A **(B)** Summary statistics are displayed as mean +/- SEM. Means compared by unpaired t-test. Despite individual and run to run variability, cells synchronized with human serum from either young (N=7 subjects) or old (N=8 subjects) individuals showed similar amplitude (top, left), period (top, right), and phase (bottom) of BMAL1-luciferase rhythms. Data points represent individual replicates within an experiment. Wells run in the same experiment are displayed in the same color. Each subject's sample was run in 2-3 replicates.

bioRxiv preprint doi: https://doi.org/10.1101/2023.04.19.537477; this version posted April 24, 2023. The copyright holder for this preprint (which was not certified by peer review) is the author/funder, who has granted bioRxiv a license to display the preprint in perpetuity. It is made available under aCC-BY 4.0 International license.

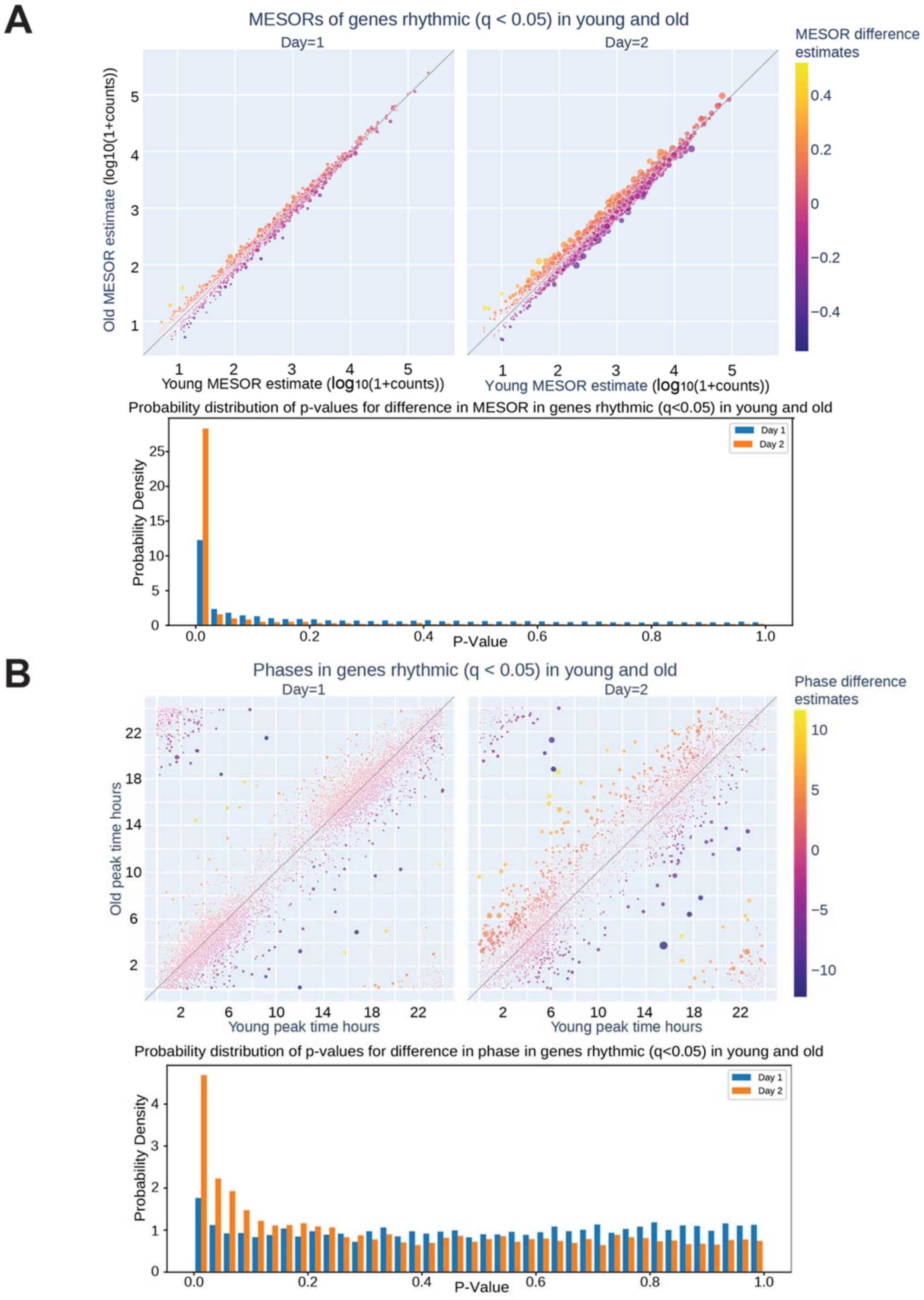

Fig S3. The circadian transcriptomes of cells synchronized with young or old sera deviate significantly on day two.

bioRxiv preprint doi: https://doi.org/10.1101/2023.04.19.537477; this version posted April 24, 2023. The copyright holder for this preprint (which was not certified by peer review) is the author/funder, who has granted bioRxiv a license to display the preprint in perpetuity. It is made available under aCC-BY 4.0 International license.

On day two of serum entrainment, young and old transcriptional rhythms differentiated. CircaCompare analysis of RNA sequencing revealed that on Day 2 of serum entrainment analysis (CT 36-58) the MESOR differences of cycling genes are larger between the cells entrained with young or old serum **(A**, top**)**. Additionally, more genes were phase shifted in the old serum condition compared to young serum on Day 2 **(B**, top**)**. The distribution of p-values shows an enrichment of low p-values on Day 2 for both MESOR and phase differences (A, bottom, B, bottom). The size of each circle in the scatter plots is proportional to $-\log_{10}q$, so the bigger the circle, the smaller the q-value for the difference between young and old for each metric.

bioRxiv preprint doi: https://doi.org/10.1101/2023.04.19.537477; this version posted April 24, 2023. The copyright holder for this preprint (which was not certified by peer review) is the author/funder, who has granted bioRxiv a license to display the preprint in perpetuity. It is made available under aCC-BY 4.0 International license.

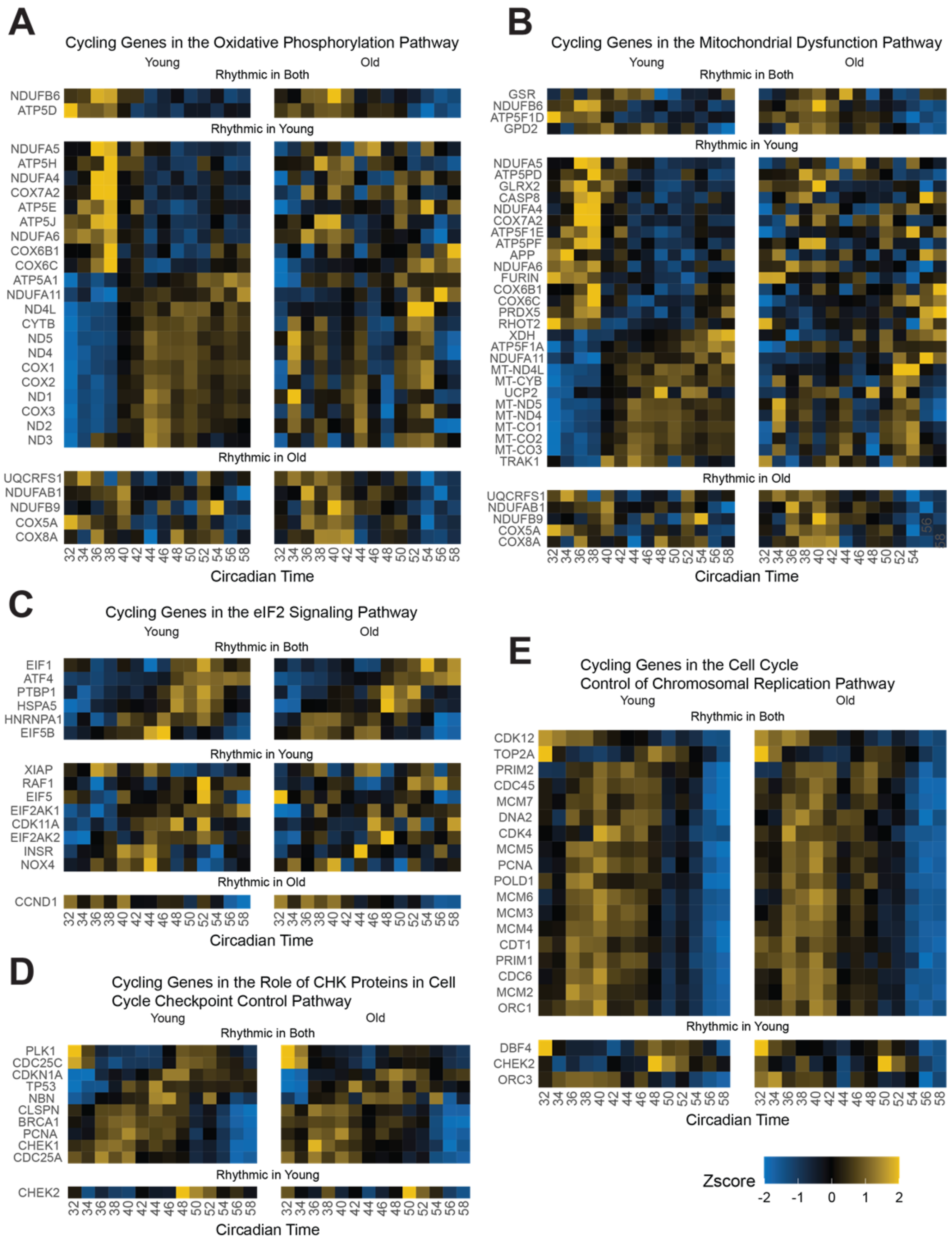

bioRxiv preprint doi: https://doi.org/10.1101/2023.04.19.537477; this version posted April 24, 2023. The copyright holder for this preprint (which was not certified by peer review) is the author/funder, who has granted bioRxiv a license to display the preprint in perpetuity. It is made available under aCC-BY 4.0 International license.

Fig S4: Oxidative phosphorylation genes and chromosomal replication genes are affected by entrainment with old serum

Additional pathway analysis employing IPA further emphasizes finding with STRING analysis. Genes identified by IPA analysis to be associated with oxidative phosphorylation/mitochondrial dysfunction and eIF2 genes lose rhythmicity with age (BIC >0.75) **(A, B, C)**. Cell cycle checkpoint control (CHK proteins) **(D)** and chromosomal replication genes **(E)** maintain their cycling in the aged condition, which is consistent with the continued division of cells.

bioRxiv preprint doi: https://doi.org/10.1101/2023.04.19.537477; this version posted April 24, 2023. The copyright holder for this preprint (which was not certified by peer review) is the author/funder, who has granted bioRxiv a license to display the preprint in perpetuity. It is made available under aCC-BY 4.0 International license.

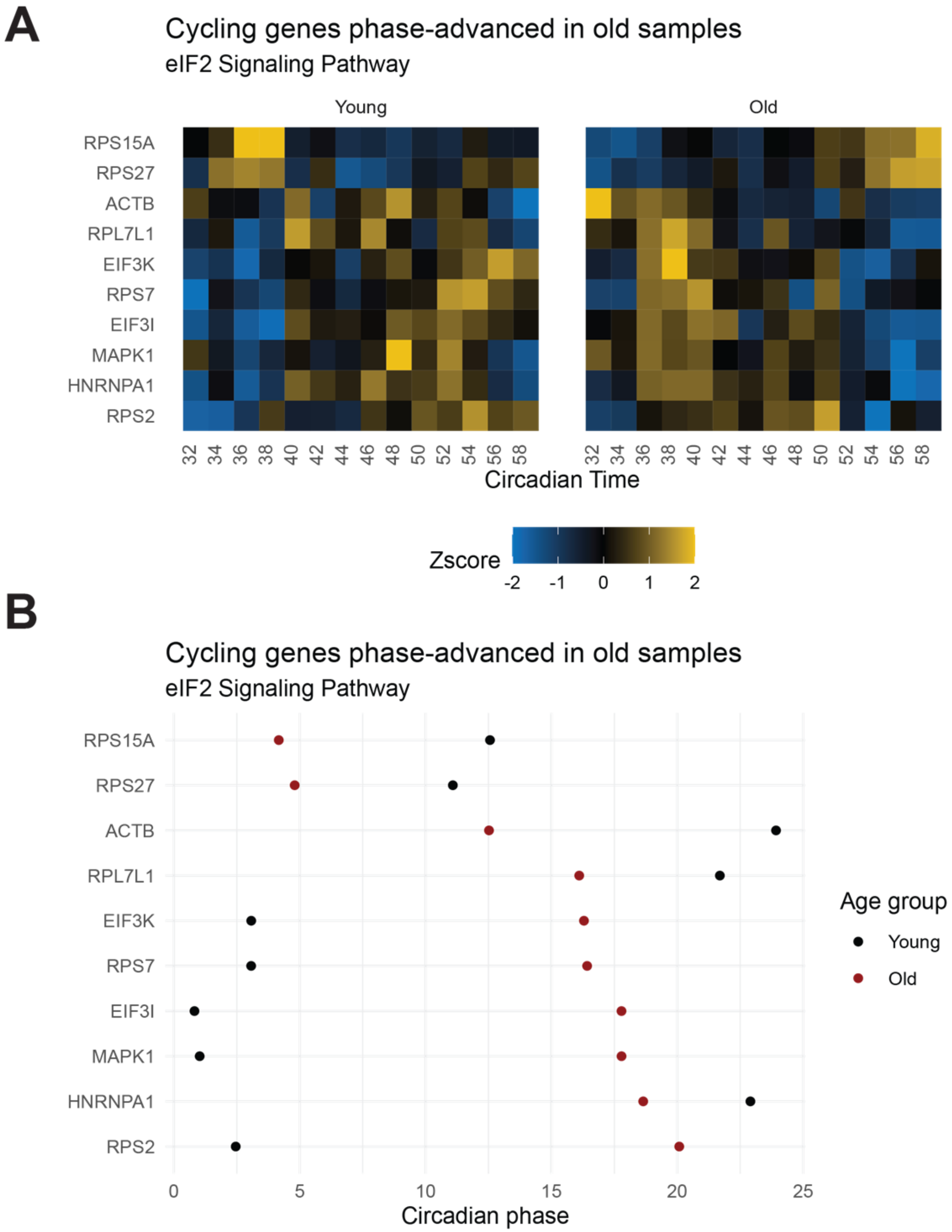

bioRxiv preprint doi: https://doi.org/10.1101/2023.04.19.537477; this version posted April 24, 2023. The copyright holder for this preprint (which was not certified by peer review) is the author/funder, who has granted bioRxiv a license to display the preprint in perpetuity. It is made available under aCC-BY 4.0 International license.

Fig S5: eIF2 signaling pathway genes that maintain cycling with age phase advance in the old serum condition

From the IPA analyses, the eIF2 signaling pathway was enriched (BIC >0.75) in two datasets: 1) genes cycling in the young sera and not the old and 2) cycling genes phase-advanced in the old sera ($q<0.05$). A significant number of genes that maintain rhythmicity in the old sera condition are phase advanced as visualized in the heat map **(A)** or plot of peak phase **(B)**. As the eIF2 pathway is involved in protein translation, this suggests that the timing of protein translation is phase advanced in the older serum and/or less rhythmic.

bioRxiv preprint doi: https://doi.org/10.1101/2023.04.19.537477; this version posted April 24, 2023. The copyright holder for this preprint (which was not certified by peer review) is the author/funder, who has granted bioRxiv a license to display the preprint in perpetuity. It is made available under aCC-BY 4.0 International license.

Gene	Symbol	Old_mesor_estimate	Young_mesor_estimate
ENSG00000014138	POLA2	1.942220257	2.030676546
ENSG00000062822	POLD1	2.727523056	2.828026817
ENSG00000073111	MCM2	3.003984606	3.115331295
ENSG00000076003	MCM6	3.124116273	3.203138512
ENSG00000085840	ORC1	2.177440998	2.305154417
ENSG00000091651	ORC6	2.415062674	2.513336383
ENSG00000093009	CDC45	2.428834807	2.540049538
ENSG00000094804	CDC6	2.566892644	2.679401971
ENSG00000097046	CDC7	2.279003902	2.377425914
ENSG00000100297	MCM5	3.17577594	3.288560637
ENSG00000101868	POLA1	2.640718445	2.728951631
ENSG00000104738	MCM4	3.310670684	3.413600036
ENSG00000112118	MCM3	3.168296215	3.269321473
ENSG00000117748	RPA2	2.754645539	2.798914718
ENSG00000131747	TOP2A	3.722304752	3.798890616
ENSG00000132383	RPA1	3.238584015	3.287556646
ENSG00000132646	PCNA	3.232760966	3.321439626
ENSG00000135446	CDK4	3.143303063	3.172936739
ENSG00000138346	DNA2	2.113453067	2.238476228
ENSG00000146143	PRIM2	2.620994795	2.675363852
ENSG00000164815	ORC5	2.361538047	2.414675015
ENSG00000166508	MCM7	3.381394362	3.482444492
ENSG00000167258	CDK12	3.10241713	3.129181459
ENSG00000167513	CDT1	2.474002764	2.590319547
ENSG00000198056	PRIM1	2.116803844	2.231962095

Table S2: Genes with decreased MESOR in the IPA cell cycle control of chromosomal replication pathway

Table of genes that are rhythmic in both conditions with a decreased MESOR with old serum treatment compared to young serum treatment by CircaCompare. All genes are involved in the cell cycle/DNA replication pathway.

bioRxiv preprint doi: https://doi.org/10.1101/2023.04.19.537477; this version posted April 24, 2023. The copyright holder for this preprint (which was not certified by peer review) is the author/funder, who has granted bioRxiv a license to display the preprint in perpetuity. It is made available under aCC-BY 4.0 International license.

Gene	Symbol	Old_mesor_estimate	Young_mesor_estimate
ENSG00000001630	CYP51A1	2.040377336	1.871207095
ENSG00000079459	FDFT1	3.330291993	3.19001773
ENSG00000104549	SQLE	3.116822033	2.950386727
ENSG00000110921	MVK	2.530172998	2.4121956
ENSG00000112972	HMGCS1	2.93111747	2.696086286
ENSG00000113161	HMGCR	3.011982868	2.833316048
ENSG00000116133	DHCR24	3.506294106	3.343492054
ENSG00000120437	ACAT2	2.71791125	2.58109012
ENSG00000132196	HSD17B7	1.940436315	1.782521965
ENSG00000147383	NSDHL	2.540302415	2.468852126
ENSG00000160285	LSS	3.486802994	3.411431601
ENSG00000160752	FDPS	3.143592562	3.081359846
ENSG00000167508	MVD	2.819963909	2.68386468
ENSG00000172893	DHCR7	2.990620862	2.797567922

Table S3: Genes with increased MESOR in the IPA cholesterol biosynthesis pathway

Table of genes that are rhythmic in both conditions with a decreased MESOR with old serum treatment compared to young serum treatment by CircaCompare. All genes are involved in the cholesterol biosynthesis pathway.
